# Excision of a Lipoma Between the Parotid Gland and Masseter Muscle via the High Perimandibular Approach: A Case Report

**DOI:** 10.7759/cureus.86706

**Published:** 2025-06-24

**Authors:** Hideharu Nakamura, Takaya Makiguchi, Nana Tomaru, Satoshi Yokoo

**Affiliations:** 1 Department of Oral and Maxillofacial Surgery, and Plastic Surgery, Gunma University Graduate School of Medicine, Maebashi, JPN

**Keywords:** cheek subcutaneous tumor, faciala nerve, high perimandibular approach, lipoma, parotid duct

## Abstract

Subcutaneous tumors in the cheek region present a surgical challenge due to the complex anatomy involving the facial nerve and parotid duct. We report the case of a 72-year-old male with a soft, mobile mass in the left subcutaneous cheek. Magnetic resonance imaging revealed a well-defined 3 cm lipomatous lesion situated between the parotid gland and the masseter muscle. The mass was hyperintense on T1- and T2-weighted images and hypointense on fat-suppressed T2-weighted imaging, consistent with a lipoma. The tumor was successfully excised en bloc using the high perimandibular approach, which is a transmasseteric technique originally developed for mandibular condylar fractures. A 5 cm incision was placed just inferior to the mandibular border, and dissection was performed between the masseter and parotid gland under facial nerve monitoring. No injury to the facial nerve or parotid duct occurred. The postoperative course was uneventful, with no complications such as facial nerve palsy or salivary leakage. Histopathological examination confirmed the diagnosis of lipoma. The surgical scar was well concealed, and there was no recurrence at 4 months. This case shows that the high perimandibular approach provides excellent access for resection of subcutaneous cheek tumors located in the central to posterior masseteric region, while minimizing the risk of injury to the facial nerve and parotid duct, and achieving a favorable cosmetic outcome.

## Introduction

The cheek region has a complex anatomy, comprising the skin, adipose tissue, parotid gland, branches of the facial nerve, and lymphatic structures, making it a site where various types of tumors can develop. Tumors located in the deep subcutaneous layer, particularly those adjacent to the masseter muscle, require careful differential diagnosis to distinguish between entities such as neurogenic tumors and accessory parotid gland tumors. Surgical resection in this anatomically dense area carries inherent risks, including facial nerve palsy and salivary leakage [1,2]. Several surgical approaches have been described for excision of subcutaneous cheek tumors, including direct skin incisions over the lesion, intraoral incisions, preauricular S-shaped incisions, submandibular incisions, and facelift-type incisions. Each technique has its advantages and disadvantages with respect to surgical access, surgical exposure, risk of complications, and postoperative cosmetic outcomes [3,4].

The high perimandibular approach, which was originally developed for treatment of mandibular condylar fractures, is a transmasseteric technique that offers a wide operative field extending from the mandibular ramus to the condylar neck [5]. This approach enables effective exposure while minimizing the risk of facial nerve injury [6, 7]. Moreover, the resulting surgical scar is well-concealed beneath the mandibular border, offering a favorable cosmetic outcome [6-8]. Although originally developed for mandibular surgery, this technique is also considered to be a useful approach for subcutaneous tumors in the cheek region. Here, we report a case of a subcutaneous lipoma located between the parotid gland and the masseter muscle that was successfully resected using the high perimandibular approach, resulting in favorable surgical and aesthetic outcomes.

## Case presentation

**Figure 1:**
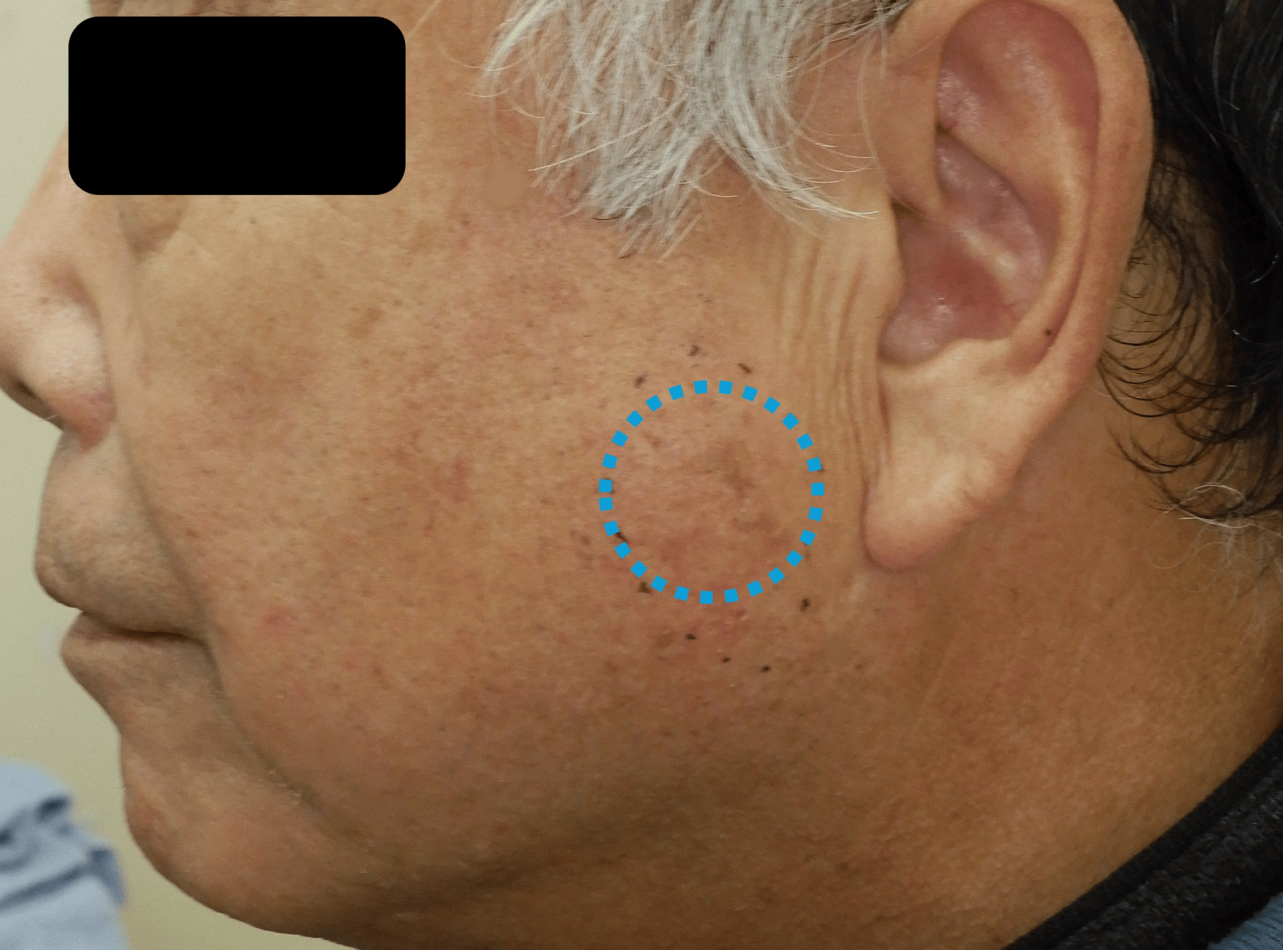
Preoperative clinical photograph. Preoperative clinical photograph of a 72-year-old male presenting with a soft, mobile mass in the left subcutaneous cheek region. The tumor is indicated by blue dotted lines.

**Figure 2:**
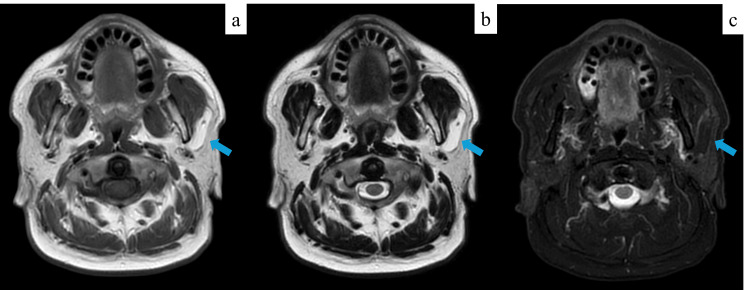
Preoperative magnetic resonance imaging. (a) T1-weighted and (b) T2-weighted images showing a well-defined 3 cm mass with high signal intensity located between the parotid gland and masseter muscle. (c) Fat-suppressed T2-weighted image showing a lesion with low signal intensity, consistent with a lipoma. The tumor is indicated by blue arrows in all images.

A 72-year-old male presented with a soft, mobile mass in the left subcutaneous cheek region, which had gradually increased in size over the past year without associated neurological or salivary symptoms (Figure 1). Given the anatomical location of the mass, differential diagnoses such as an intramasseteric tumor or a parotid gland tumor were considered. Magnetic resonance imaging (MRI), used for further evaluation as it is the most appropriate modality for evaluating the spatial relationship between the tumor and surrounding soft tissues, as well as for qualitative assessment, showed a well-circumscribed, 3 cm lesion located between the parotid gland and the masseter muscle. The mass exhibited high signal intensity on both T1- and T2-weighted images and low signal intensity on fat-suppressed T2-weighted imaging, consistent with a lipoma (Figure 2).

Surgical technique 

**Figure 3:**
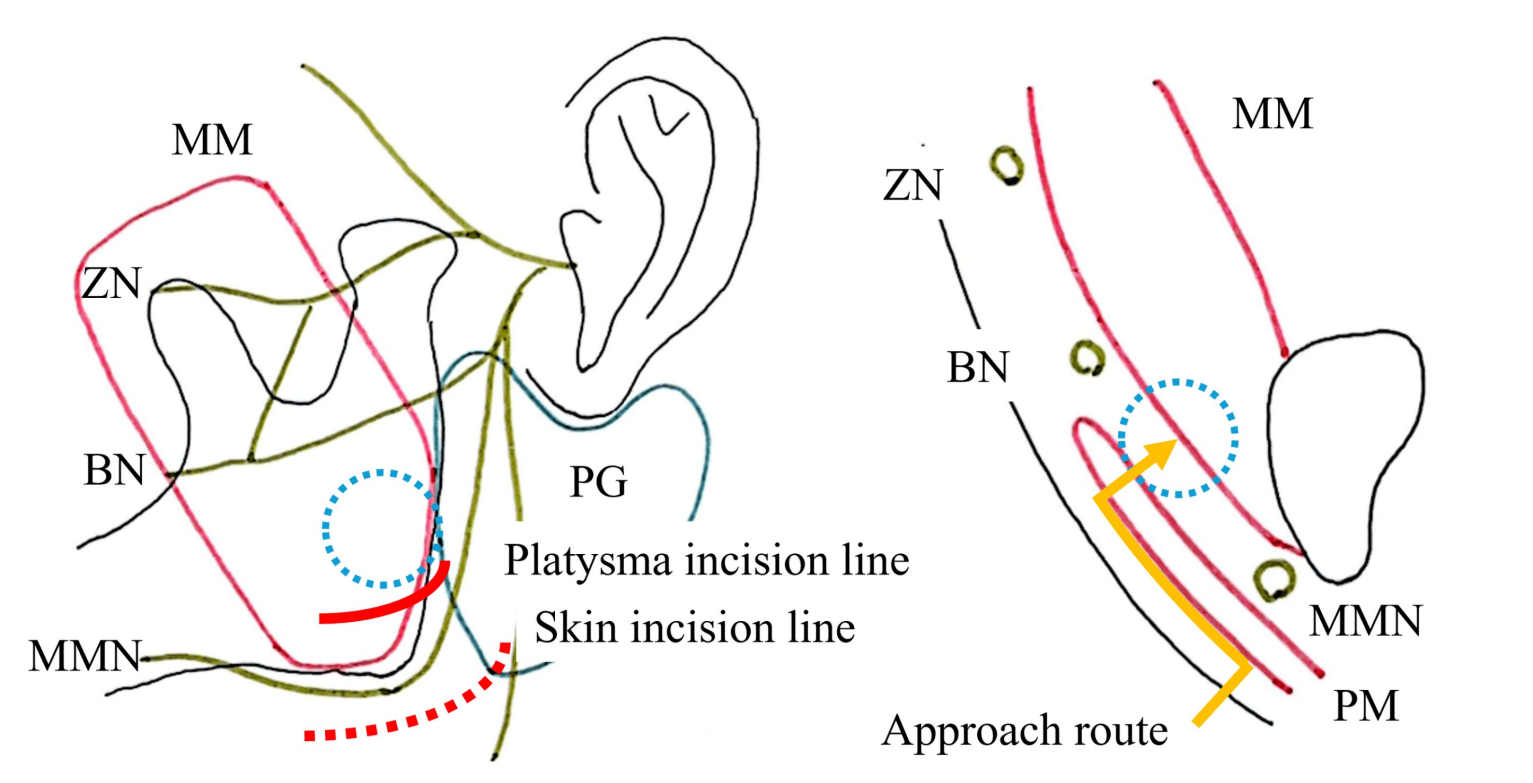
Schematic illustration of the high perimandibular approach. The tumor is indicated by blue dots. The red dotted line represents the skin incision, and the red solid line indicates the platysma incision. The yellow solid line depicts the surgical approach route in the frontal cross-sectional view. MM: masseter muscle; PM: platysma muscle; PG: parotid gland; ZN: zygomatic branch of the facial nerve; BN: buccal branch of the facial nerve; MMN: marginal mandibular branch of the facial nerve. This figure was created by the authors and is original.

**Figure 4:**
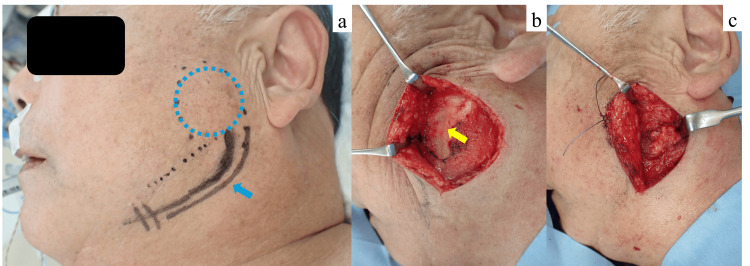
Intraoperative views of the high perimandibular approach. (a) A 5cm skin incision was made 5 mm inferior to the mandibular border and parallel to the angle of the mandible. The tumor is indicated by blue dots, and the skin incision line is indicated by blue arrows. (b) The skin and subcutaneous tissue were elevated over the platysma muscle, which was then incised approximately 1 cm superior to the mandibular border, beyond the course of the marginal mandibular branch. The yellow arrow indicates the site of platysma incision. (c) Dissection was performed between the masseter muscle and the parotid gland under facial nerve monitoring to avoid nerve injury.

**Figure 5:**
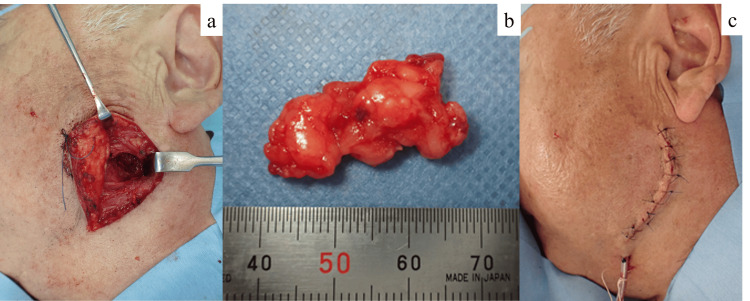
Intraoperative and immediate postoperative views. (a) The tumor was identified and excised en bloc. (b) The excised tumor specimen. (c) A closed-suction drain was placed and the wound was closed in layers.

Under general anesthesia, a 5-cm skin incision was made 5 mm inferior to the mandibular border and parallel to the angle of the mandible (Figure 3). Following subcutaneous infiltration with 1% lidocaine with 1:100,000 epinephrine, the skin was incised using a No. 15 scalpel. The skin and subcutaneous tissue were dissected and elevated over the platysma muscle using scissors. The platysma was then incised approximately 1 cm superior to the mandibular border, beyond the course of the marginal mandibular branch of the facial nerve (MMN). Blunt dissection was carefully performed between the masseter muscle and the parotid gland under continuous facial nerve monitoring to avoid injury to the buccal branch of the facial nerve (BN) (Figure 4). The tumor was identified and excised en bloc. After copious irrigation and hemostasis, a 10 Fr closed-suction drain (J-VAC, Ethicon, USA) was placed beneath the platysma. Layered wound closure was performed using 3-0 Vicryl (polyglactin 910, Ethicon, USA) for the platysma and subcutaneous tissues, 4-0 PDS II (polydioxanone, Ethicon, USA) for the dermis, and 5-0 Ethilon (nylon, Ethicon, USA) for the skin (Figure 5). The total blood loss was minimal, and the operative time was 1 hour and 50 minutes.

Postoperative course

**Figure 6:**
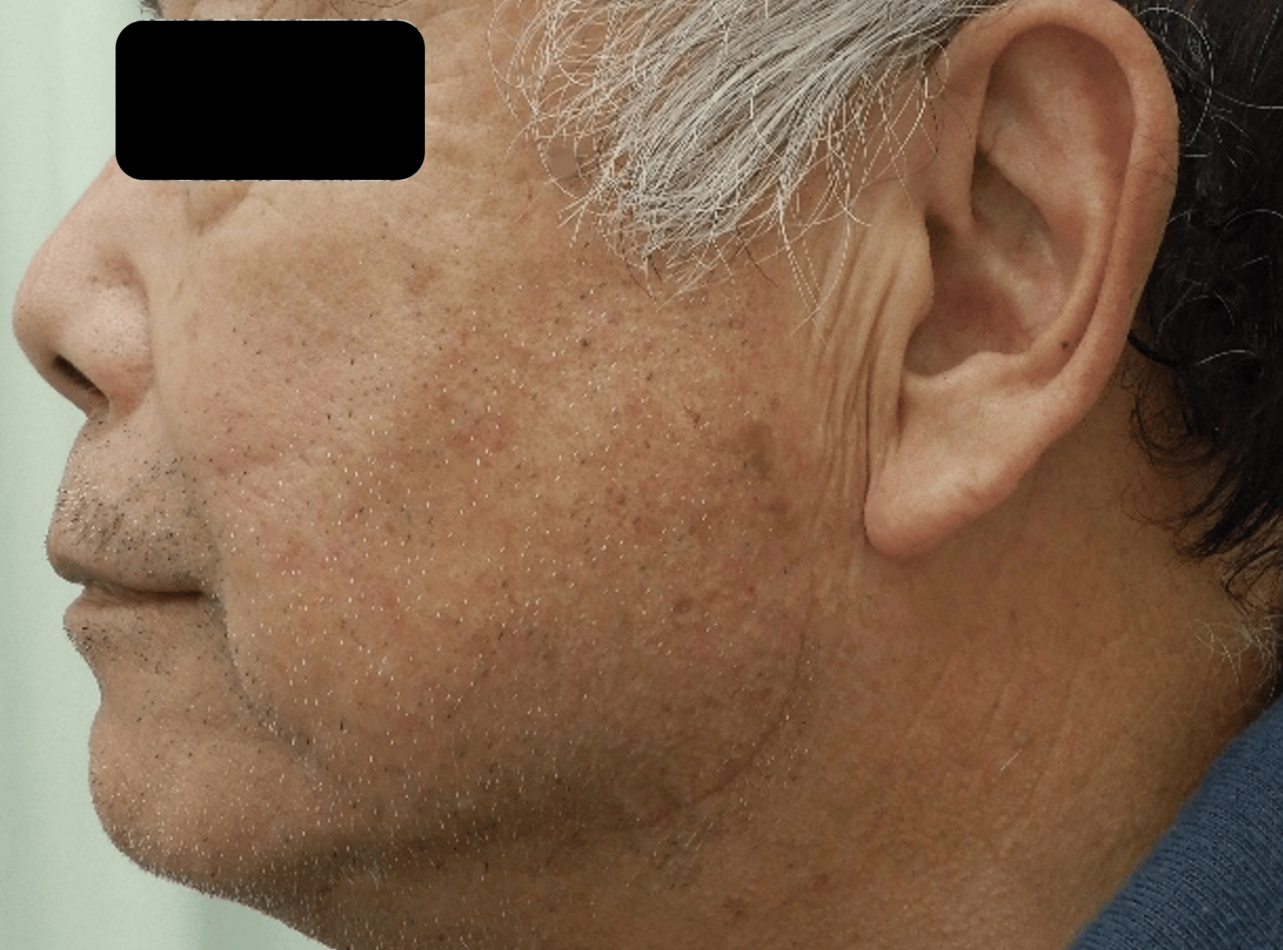
Postoperative follow-up at 4 months. The surgical scar is well concealed beneath the mandibular margin. There was no evidence of tumor recurrence or postoperative complications such as facial nerve palsy or salivary leakage.

The patient had an uneventful postoperative course. Histopathological examination confirmed the diagnosis of lipoma. No facial nerve palsy, salivary leakage, or other complications were observed. At 4 months follow-up, the scar was well concealed under the mandibular margin, and there was no evidence of tumor recurrence (Figure 6).

## Discussion

Cheek tumors located deep in the subcutaneous tissue and adjacent to the masseter muscle present significant surgical challenges due to their proximity to the facial nerve and parotid duct. Various surgical approaches have been described for accessing this region, including direct skin incisions, preauricular S-shaped incisions, submandibular incisions, intraoral approaches, and facelift-type incisions. Each of these techniques has specific advantages and disadvantages regarding surgical access, surgical exposure, risk of complications, and postoperative cosmetic outcomes [3,4].

Direct skin incisions over the lesion allow for a straightforward and minimally invasive approach; however, the limited operative field may increase the risk of injury to the facial nerve and parotid duct when dissecting deep structures. Additionally, the resulting scars on the cheek may be a concern cosmetically. Preauricular S-shaped and submandibular incisions provide broader access, but are associated with a higher risk of facial nerve injury. The reported rates of postoperative facial nerve palsy following masseteric hemangioma resection are about 29% with preauricular S-shaped incisions and up to 45% with submandibular incisions [9]. Thus, a preauricular S-shaped incision, which requires dissection of the facial nerve, and a submandibular incision, which involves superior traction of the MMN, carry a high risk of facial nerve injury. Other reported approaches include intraoral incisions and facelift-type incisions. The intraoral approach offers the advantage of avoiding external scarring; however, it provides limited surgical exposure and is generally indicated only for lesions located at the anterior border of the masseter muscle. Facelift-type incisions provide excellent aesthetic outcomes and allow visualization of both the facial nerve and parotid duct, thereby minimizing the risk of injury. However, this technique requires extensive subcutaneous dissection and is associated with greater surgical invasion.

Anatomically, the zygomatic branch of the facial nerve (ZN) and BN frequently form cross-communications over the masseter muscle (70-100%), while the MMN has a lower communication rate with other branches (0-16%) [7]. The MMN arises within the parotid gland and typically courses forward and anteriorly toward the angle of the mandible, running deep to the platysma. It may wind around the inferior border of the mandible and ascend to innervate the depressor anguli oris and depressor labii inferioris muscles [10]. Injury to the MMN can result in lower lip asymmetry during facial expression, particularly noticeable while smiling or speaking. Anatomical reviews have demonstrated that over 50% of MMN have multiple rami, and their position relative to the mandibular border shows considerable variation, ranging from 1.6 cm above to 2.5 cm below the margin [11]. Aravena et al. reported that the maximum distance from the MMN to the mandibular border may reach 4.01 cm, with a mean of 1.64 ± 0.92 cm [12]. These findings indicate that the traditional recommendation to place submandibular incisions at least 2 cm below the mandibular margin may not always be sufficient to avoid nerve injury. Moreover, subplatysmal dissection, which is often required in such approaches, places the dissection plane at the level of the MMN, increasing the risk of injury, especially with upward traction or blunt dissection [13].

The high perimandibular approach is a transmasseteric technique used to access the mandibular condylar process via an incision placed below the mandibular border [5]. In this approach, the skin incision is placed just inferior to the mandibular border, and the platysma is incised approximately 1 cm superior to this border. This allows for safe dissection while preserving the MMN, which is typically not interconnected with other facial nerve branches. Intraoperative exposure of the BN may occur, but identification and preservation of the branch on the masseteric fascia are generally straightforward. Furthermore, the use of intraoperative facial nerve monitoring, as employed in the present case, can further enhance nerve safety by enabling real-time identification and protection of nerve branches. Even in the event of BN injury, the high rate of cross-communication among the ZN and BN reduces the likelihood of postoperative facial nerve dysfunction. Thus, the reported incidence of facial nerve injury with the high perimandibular approach ranges from 0% to 0.9%, supporting its safety in this regard [6,7].

The high perimandibular approach is useful for both condylar fractures and subcutaneous cheek tumors located around the masseter muscle. However, its use in this context has only been described in a few cases, including the present case involving a lipoma between the masseter and parotid gland, and previous reports involving intramasseteric hemangiomas [14]. Based on these reports and the surgical field provided, the high perimandibular approach seems to be particularly useful for tumors located in the central to posterior region of the masseter muscle. However, further accumulation of cases is necessary to fully evaluate the indications, safety, and long-term outcomes of this approach in tumor surgery.

## Conclusions

The high perimandibular approach is a useful surgical option for resection of subcutaneous cheek tumors located in the central to posterior region of the masseter muscle. This approach offers sufficient operative exposure while minimizing the risk of injury to the facial nerve and parotid duct, and yields favorable cosmetic outcomes.
